# ACC Deaminase Producing *Methylobacterium oryzae* CBMB20 and Exogenous Trehalose Application Alleviate Salinity Stress in *Arabidopsis*

**DOI:** 10.4014/jmb.2501.01007

**Published:** 2025-07-11

**Authors:** Kiyoon Kim, Ei Phyu Kyaw, Krishnamoorthy Ramasamy, Denver I. Walitang, Raghu Rajasekaran

**Affiliations:** 1 National Forest Seed Variety Center, Chungju 27495, Republic of Korea; 2 Department of Biotechnology Research, Ministry of Science and Technology, Kyaukse 05151, Mandalay Region, Myanmar; 3 Chey Institute for Advanced Studies, Gangnam-gu, 06141, Seoul, Republic of Korea; 4 Department of Crop Management, Vanavarayar Institute of Agriculture, Pollachi - 642 103, Tamil Nadu, India; 5 DNA Barcoding Laboratory and College of Arts and Sciences, Romblon State University, Romblon 5505, Philippines; 6 Department of Plant Biotechnology, Centre for Plant Molecular Biology and Biotechnology, Tamil Nadu Agricultural University, Coimbatore – 641 003, Tamil Nadu, India

**Keywords:** *Arabidopsis thaliana*, *Methylobacterium oryzae*, PGPR, salt stress, trehalose, osmolytes

## Abstract

Farming communities are very concerned about salt stress because of its negative impact on crop productivity. This study evaluated the ability of the *Methylobacterium oryzae* CBMB20 and exogenous trehalose treatments on *Arabidopsis thaliana* growth under salt stress conditions. *A. thaliana* growth was enhanced using *M. oryzae* CBMB20 as a bioinoculant in both saline and non-saline environments. In addition to better photosynthetic efficiency and endogenous trehalose content, the inoculation of *M. oryzae* CBMB20 produced improved growth parameters, such as increased rosette fresh weight and shoot length. Reduced levels of proline and malondialdehyde (MDA) under salt stress (150 mM NaCl) further indicated that the inoculated plants had enhanced tolerance to salinity. GFP-tagged *M. oryzae* CBMB20 was also used in spatial distribution experiments, which showed that the bacteria colonized *A. thaliana*'s root, shoot, and hypocotyl. By increasing shoot length and total fresh weight, the exogenous application of trehalose also markedly enhanced plant growth. Proline and MDA contents were decreased by exogenous trehalose during salt stress, while the endogenous trehalose concentration in *A. thaliana* remained unaffected. The use of trehalose and *M. oryzae* CBMB20 can both have a good impact on plant development and stress tolerance in saline environments.

## Introduction

Plants are routinely exposed to a range of abiotic stimuli during their life cycle, such as heat, cold, salt, drought, and heavy metals. These stresses have a negative impact on plants' development, growth, and yield [1]. One significant abiotic stressor that has a global impact on agricultural productivity and food security is soil salinity. Twenty percent of irrigated lands, or over 62 million hectares, are currently affected by excessive salinity, and by 2050, it is predicted to affect more than 50% of arable areas [2]. Many plants become susceptible to soil salinization, which lowers turgor, reduces harvest quantity and quality, and slows down growth and development rates. Every important aspect of plant life is impacted by salt stress, including germination, growth, pigments involved in photosynthetic processes, water relations, nutritional balance, oxidative stress, and yield. Therefore, methods such as soil flushing, liming, plant breeding and genetic engineering of salt tolerant crops are employed to alleviate soil salinity. However, these strategies can lead to the genetic erosion of native crop species, are time consuming, expensive, and have limited success [3]. Plants also employ various strategies to alleviate salt stress with the help of endosphere, phyllosphere, and rhizosphere microbiomes [4].

Harnessing the plant microbiome to improve productivity and overcome multiple stresses is a key to sustainable crop production [5]. Plant growth-promoting rhizobacteria (PGPR) are recognized for alleviating both abiotic and biotic stresses encountered by plants [6, 7]. Plant growth is significantly improved by PGPR through direct and indirect mechanisms [8]. Directly, they enhance plant growth by regulating phytohormone levels, increasing siderophore production, and improving nutrient availability through nitrogen fixation and phosphorus/ion solubilization [9]. Indirectly, PGPR promote plant growth by enhancing plant tolerance to both biotic and abiotic stresses [10]. The use of PGPR in agriculture has been recommended to achieve sustainable and eco-friendly crop production, particularly for stress alleviation [11]. PGPR like *Azospirillum*, *Arthrobacter*, *Azotobacter*, *Bacillus*, *Burkholderia*, *Enterobacter*, *Pseudomonas*, and *Methylobacterium oryzae* have been demonstrated to enhance salt tolerance in various crops [12-14]. These bacteria can reduce the toxic effects of high salinity by increasing biomass, chlorophyll content, and the production of osmoprotectants such as proline and malondialdehyde (MDA) [15].

Several ACC deaminase-producing halotolerant bacteria isolated from coastal saline soils are reported to have plant growth-promoting activities under salinity [16]. The occurrence of the genus *Methylobacterium* has been reported in more than 70 plant species either as free living, as endophytic, or as epiphytic symbiotic bacteria. *Methylobacterium oryzae* CBMB20 is a pink-pigmented bacterium that produces 1-aminocyclopropane-1-carboxylate deaminase (ACCD) [17, 18], along with indole-3-acetic acid (IAA), extracellular polysaccharides (EPS), polyhydroxybutyrate and biofilm [19]. According to Wang [20], inoculating ACC deaminase producing bacteria enhances the photosynthetic characteristics of salt-stressed pea plants. *M. oryzae* CBMB20 have been reported to enhance plant growth and productivity through enhanced nutrient accumulation [21, 22] and production of phytohormone [23, 24]. Rice cultivars treated with *M. oryzae* CBMB20 showed improved plant vacuolar H+ ATPase activity, and photosynthetic characteristics and decreased ACC accumulation, ACO activity and VOC emission there by the effect of salinity was alleviated [25]. Apart from conferring ACC deaminase-mediated salinity tolerance, *M. oryzae* CBMB20 also induces defense responses in tomatoes [26, 27].

Under salt stress, osmolytes like glycine betaine, proline, and trehalose can help maintain cell turgor, stabilize proteins, scavenge reactive oxygen species (ROS), and protect membranes from oxidative damage. Therefore, it has been proposed that applying osmolytes externally, either individually or in combination, is an effective strategy for mitigating the effects of salt stress in plants [28]. Exogenous application of trehalose has shown promising potential for crop improvement. For instance, low concentrations of trehalose can decrease sodium ion accumulation in plants, while higher concentrations can prevent chlorophyll loss in leaves and root damage caused by high salt levels, thereby alleviating salt-induced damage [29, 30]. Additionally, research has shown that exogenous trehalose significantly affects ion imbalance, ROS proliferation, and programmed cell death induced by salt stress in *Arabidopsis thaliana* [31], maize [32], *Catharanthus roseus* [33] and tomato [28]. Furthermore, trehalose significantly decreased MDA accumulation in tomato, suggesting that it can reduce cell membrane damage and enhance salt tolerance in the plant [28]. However, report on ACC deaminase producing bacteria and in combination with trehalose on salt stress alleviation is scanty. This study aimed to assess the effects of ACC deaminase producing *M. oryzae* CBMB20 and the application of trehalose on *Arabidopsis thaliana*, specifically focusing on their potential to alleviate salt stress. Key aspects investigated include plant growth, proline content, and levels of chlorophyll a (Chl a), chlorophyll b (Chl b), carotenoids, MDA, and trehalose sugar production.

## Materials and Methods

### Plant Material and Salt Tolerance Assay

The wild type *Arabidopsis thaliana* Columbia 0 ecotype (Col-0 accession) seeds were surface disinfected for 5 min in 30% NaOCl and rinsed well with sterile water. The surface disinfected seeds were re-suspended in sterile water and stored at 4°C in the dark for 72 h. The seeds were then placed on 0.4% agar plates containing ½ strength MS medium + 0.5% sucrose (pH 5.7). The plates were incubated in growth chamber at 22 ± 2°C with 16 h light/8 h dark photoperiods, at a light intensity (photon flux density) of 99.5 μmole m^-2^s^-1^. In the experiment, 10-day-old Col-0 seedlings were transplanted into plastic plots (dimensions: inner diameter = 9 cm; outer diameter = 10 cm; depth = 9 cm) containing 100 g nursery soil (Nongwoo-Bio Co., Ltd., Republic of Korea; composition: 58.8% coco peat, 17% peat moss, 10% perlite, 10% vermiculate, 4% zeolite, 0.004% pyroligneous acid and 0.01% wetting agent) and grown in a growth chamber. Salt stress was induced gradually by applying 25 mM of sodium chloride solution to each pot on alternative days to avoid osmotic shock; and the desired salt concentrations of 50, 100, 150 and 200 mM were achieved after 2, 6, 10, and 14 days, respectively. Preliminary tests revealed that *A. thaliana* plants can tolerate up to 150 mM NaCl, but at 200 mM NaCl, the stress symptoms become quite severe such as retarded growth, leaf senescence and loss of turgor. After 14 days of NaCl treatment, the morphology of the plants was observed, and the physiological indices were measured to determine the optimal NaCl concentration. Based on the results obtained in this study, 150 mM NaCl was chosen for further experiments.

### Microbial Culture and Growth Conditions

*Methylobacterium oryzae* CBMB20 (obtained from Department of Agricultural Chemistry, Chungbuk National University, Republic of Korea) used in this study was maintained in ammonium mineral salts (AMS) medium (K_2_HPO_4_ 0.7 g/l; KH_2_PO_4_ 0.54 g/l; MgSO_4_·7H_2_O 1.0 g/l; CaCl_2_·2H_2_O 0.2 g/l; FeSO_4_·7H_2_O 4.0 mg/l; ZnSO_4_·7H_2_O 100 μg/l; MnCl_2_·4H_2_O 30 μg/l; H_3_BO_3_ 300 μg/l; CoCl_2_·6H_2_O 200 μg/l; CuCl_2_·2H_2_O 10 μg/l; NiCl_2_·6H_2_O 20 μg/l; Na_2_MoO_4_·2H_2_O 60 μg/l; NH_4_Cl 0.5 g/l) supplemented with 0.5% sodium succinate as the sole carbon source at 30°C. Single colonies were inoculated in Ammonium Mineral Salts (AMS) medium broth and grown aerobically on a rotary shaker (150 rpm) at 30°C for up to 72 h. Prior to inoculation, bacterial cells were harvested by centrifugation (9,000 rpm, 10 min, 4°C), washed 3 times with sterile 30 mM MgSO_4_ to remove the growth medium and re-suspended in 30 mM MgSO_4_ to a final population density of 10^8^ CFU ml^-1^ and used for inoculation experiments.

### Microbial Treatment and Imposing Salt Stress

The effectiveness of bacterial treatment in improving plant resistance to salt stress was assessed using a modified version of the methods [34]. Briefly, uniformly developed, 10-days-old healthy *A. thaliana* seedlings were transferred aseptically from ½ strength Murashige and Skoog (MS) agar plates to pots filled with nursery soil as described previously, one seedling per pot was maintained. Each treatment included three replicates, with a total of ten plants per treatment using a completely randomized design. After three days of acclimation, the plants were root-treated with 10 ml of a bacterial suspension (~ 10^8^ CFU ml^-1^). Control *A. thaliana* plants were inoculated with sterile 30 mM MgSO_4_. Five days after bacterization, salt stress was induced gradually by applying 25 mM of sodium chloride solution to each pot on alternative days to avoid osmotic shock; and the desired salt concentrations of 150 mM was achieved after 10 days. Parallel controls were maintained by irrigating with tap water. The leaching of water from the pots was prevented by retaining the soil water to a level below water holding capacity. The soil electrical conductivity of 0, and 150 mM treatments were 1.31 and 16.23 dS/m, respectively, at the time of harvest. Fourteen days after exposing to salt stress, the plants were uprooted for growth parameter measurements.

### Exogenous Trehalose Application and Assessing Salinity Tolerance

In this experiment, the 10-days-old wild-type Col-0 seedlings were transplanted into plastic plots (dimensions: inner diameter = 9 cm; outer diameter = 10 cm; depth = 9 cm) containing 100 g nursery soil and grown in a growth chamber. To study the role of trehalose against salinity, trehalose at the concentration of 0, 5, 10 and 15 mM was applied exogenously in the roots at the rate of ten ml per plant [35]. After 14 days of treatment, the seedlings were harvested for the assay of phenotypic changes. Based on the above experiment, four treatments were set and 10 mM trehalose was chosen for the further experiment based on physical parameters of plants.

A pot culture experiment was carried out, as detailed in the preceding section, to assess the impact of trehalose on reducing salt stress. Briefly, the 10-days-old wild-type Col-0 seedlings were transplanted into plastic plots (dimensions: inner diameter = 9 cm; outer diameter = 10 cm; depth = 9 cm) containing 100 g nursery soil and grown in a growth chamber. After 5 days of exogenous trehalose application, salt stress was induced gradually by applying 25 mM of sodium chloride solution to each pot on alternative days to avoid osmotic shock; and the desired salt concentrations of 150 mM was achieved after 10 days. Control plants were watered with tap water throughout the experiment, and to prevent water leaching, soil moisture was maintained below its water-holding capacity. The plants were harvested at 14 days after NaCl treatment the plants were uprooted for growth parameter measurements.

### Phenotypic Assay and Photosynthetic Pigment Analysis

The parameters assessed included the whole fresh weight, shoot length, root length, and rosette fresh weight of *Arabidopsis*. The shoots of these seedlings were detached, rinsed with deionized water, dried, and weighed. Photosynthetic pigments (Chl a, Chl b, and carotenoids) were measured following the procedures described by Sumanta [36]. Briefly, the leaf samples (0.3 g) were homogenized in a tissue homogenizer using 80% (w/v) acetone. The homogenate was centrifuged at 6,000 × *g*, and the supernatant was used for pigment analysis. Absorbance of the extracted pigments was measured in UV-Visible Spectrophotometer (NEO-D3117, NEOGEN, Republic of Korea) at 480 nm, 510 nm, 645 nm and 663 nm. The photosynthetic pigment content was calculated using the following equations:

Chl a = [12.7 (OD_663_) -2.69 (OD_645_) × [(final volume of filtrate/1000) × 0.3)]

Chl b = [22.9 (OD_645_) -4.68 (OD_663_) × [(final volume of filtrate/1000) × 0.3)]

Carotenoid = [7.6 (OD_480_) -1.49 (OD_510_) × [(final volume of filtrate/1000) × 0.3)]

### Determination of MDA Concentration

Malondialdehyde content was measured using the thiobarbituric acid (TBA) test, in which two molecules of TBA react with MDA to form a pinkish-red chromogen [37]. 0.5 g of ground frozen tissue was combined with 5 ml of 0.1% (w/v) trichloroacetic acid (TCA) in a 2 ml microcentrifuge tube. The tubes were centrifuged at 6,000 × *g* for 10 min, and 200 μl of the supernatant was mixed with 800 μl of 0.5% (w/v) TBA in 20% (w/v) TCA. The tubes were then incubated on a dry bath for 30 min at 100°C. After incubation, the tubes were cooled on ice to halt the reaction and centrifuged for 15 min at 6,000 × *g*. The supernatant absorbance was measured at 532 and 600 nm using a UV-Vis spectrophotometer, with 0.5% (w/v) TBA in 20% (w/v) TCA used as the blank. The MDA content was calculated by subtracting A_532_ from A_600_ and multiplied by the extinction coefficient 155 mm^-1^ cm^-1^.

### Determination of Proline Concentration

Leaf samples (0.3 g) were extracted in 3% sulfosalicylic acid and centrifuged for 10 min at 6,000 × *g*. Two ml of the supernatant were incubated with an equal volume of acid ninhydrin and glacial acetic acid at 100°C for 1 h. The reaction was stopped by placing the samples in an ice bath, and proline was separated using toluene. Absorbance was then recorded at 520 nm using a UV-Vis spectrophotometer [38]. The proline content was determined using a standard curve plotted with the known concentration of L-proline and expressed as unit μmoles of proline/g fresh weight.

### Determination of Cellular Trehalose Concentration

Trehalose content in leaves was measured using the Li method [39]. One gram of leaf was homogenized in 5 ml of 80% (v/v) hot ethanol and centrifuged at 9000 rpm for 20 min. The supernatant was evaporated at 80°C and suspended in 5 ml of distilled water. A 100 μl aliquot of this solution was mixed with 150 μl of 0.2N H_2_SO_4_ and boiled at 100°C for 10 min then cooled on ice. Further, 150 μl of 0.6 N NaOH was added, and the mixture was boiled for another 10 min to destroy reducing sugars before being cooled again. Finally, 2.0 ml of anthrone reagent (0.2 g anthrone per 100 ml of 95% H_2_SO_4_) was added to the mixture, which was then boiled for 10 min to develop colour and subsequently cooled. Absorbance was measured at 630 nm, and trehalose concentration was determined using a standard curve.

### Microscopic Analysis of Bacterial Colonization by Green Fluorescence Protein (GFP)-Tagged *M. oryzae* CBMB20

Single colonies of GFP-tagged *M. oryzae* were grown in AMS medium amended with kanamycin (20 μg/ml) to mid-exponential phase at 30°C. The bacterial cell was pelleted by centrifugation (10,000 rpm, 10 min, 4°C) and washed twice in sterile 30 mM MgSO_4_. The bacterial suspension was adjusted to a population density of 10^8^ CFU ml^-1^ in sterile 30 mM MgSO_4_. For seed treatment, surface sterilized *A. thaliana* seeds were incubated in shaker at 60 rpm for 1 h in 2 ml of bacterial suspension in 30 mM MgSO_4_ and with sterile 30 mM MgSO_4_ as control. The seeds were grown on plates containing ½ MS medium for 4 days. Then seedlings with uniform size were transferred to square of a plate containing ½ MS agar with 150 mM NaCl and without NaCl. After 10 days, the plantlets were washed with phosphate buffer solution and examined under the confocal microscope.

### Statistical Analysis

The data were analyzed by an analysis of variance (ANOVA) using the general linear model version 9.1; SAS institute Inc, Cary, NC, USA. Means were compared by Tukey’s post hoc test.

## Results

### Growth Promotion of *Arabidopsis thaliana* by *M. oryzae* CBMB20

**Fig. 1:**
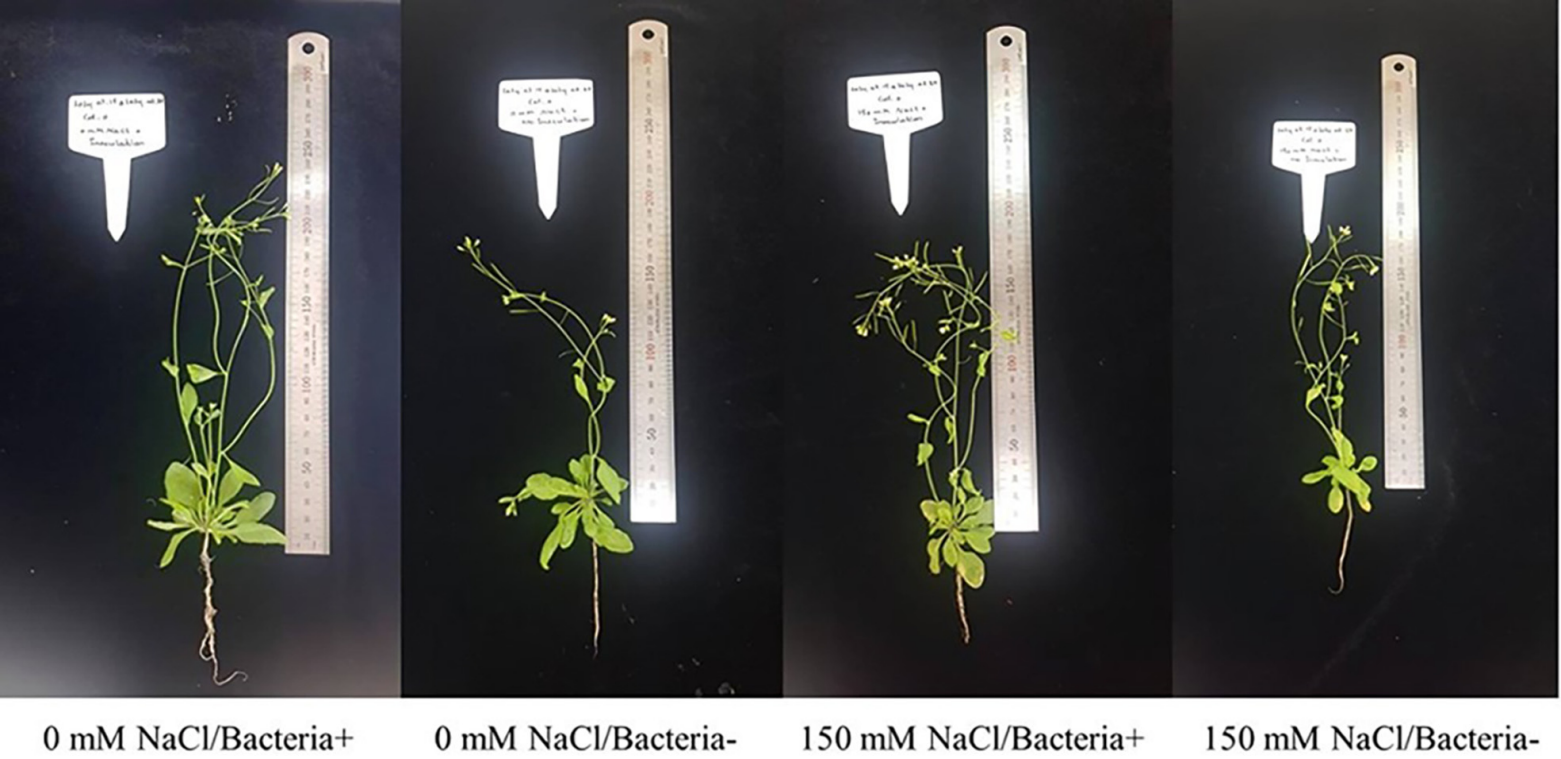
The growth of *Arabidopsis thaliana* plants after inoculation with *M. oryzae* CBMB20 under salt stress and non-stress conditions. Bacteria+ (treated with *M. oryzae* CBMB20); Bacteria- (not treated with *M. oryzae* CBMB20).

**Fig. 2:**
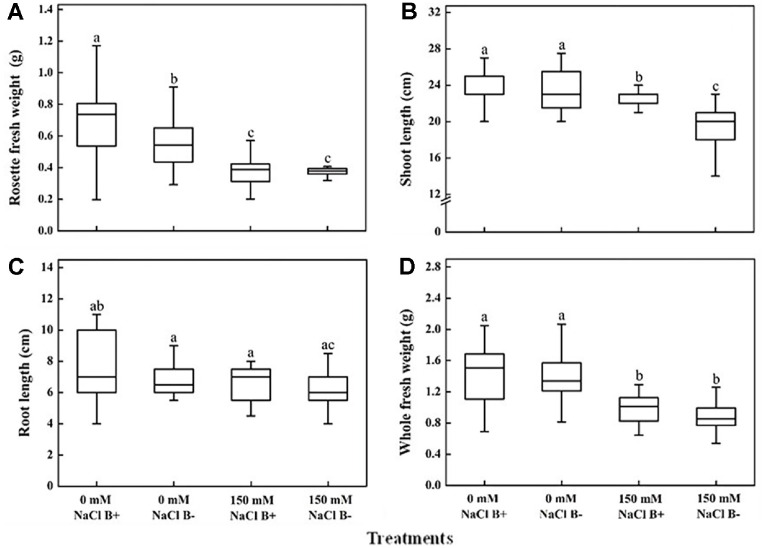
Effects of *M. oryzae* CBMB20 on plant growth under salt stress and non-stress conditions. *Arabidopsis* development phenotypes were analyzed in response to salt stress, inoculated with (B+) and without (B-) *M. oryzae* CBMB20 under 0 mM NaCl and 150 mM NaCl. Figures show (**A**) Rosette Fresh Weight, (**B**) Shoot Length, (**C**) Root Length, and (**D**) Whole Fresh Weight. Different letters indicate the significant difference among treatments (*p* < 0.05); according to one way ANOVA test; Data are the mean ± SD, *n* = 21.

In this study, the interaction between *M. oryzae* CBMB20 and *Arabidopsis* was explored under salt stress conditions and demonstrated that salt stress impaired shoot and root development. Under different levels (0–200 mM) of salt stress conditions, all plants survived. As shown in Fig. 1, bacterial inoculation reduced the symptoms of leaf chlorosis induced by salt stress. Imposing of salt stress significantly reduced the Rosette fresh weight, shoot and root lengths. However, inoculation of *M. oryzae* CBMB20 significantly improved shoot (*p* < 0.01) and root lengths under salt stress condition (Fig. 2). Even though higher fresh weight was observed in the plants treated with *M. oryzae* CBMB20 the results are not significant under salt stress (150 mM).

### Photosynthetic Pigment Changes by *M. oryzae* CBMB20

**Fig. 3:**
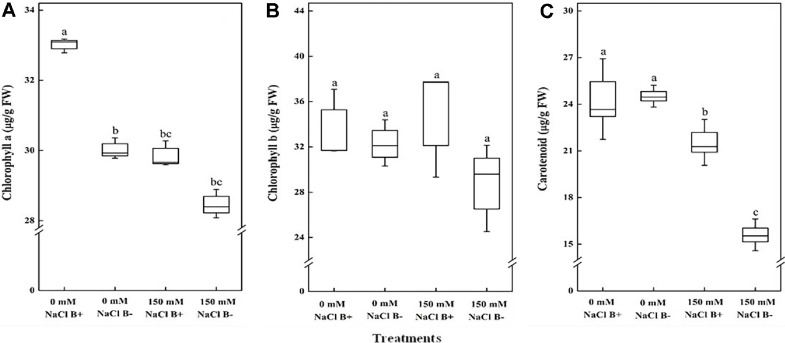
Effects of *M. oryzae* CBMB20 on photosynthetic pigments of *Arabidopsis* plants grown under salt stress and non-stress conditions. Figures show (**A**) Chlorophyll a, (**B**) Chlorophyll b, and (**C**) Carotenoid. The data represents the mean ± SD of three replicates and the different letters indicate the significant difference among treatments (*p* < 0.05); according to one way ANOVA test. Treatments details are the same as in Fig. 2.

In order to investigate the effects of *M. oryzae* CBMB20 on the photosynthetic efficiency of plants under salt stress, the leaf chlorophyll and carotenoid contents were measured. Leaf chlorophyll and carotenoid content of *Arabidopsis* plant is significantly reduced because of salt stress. The leaf chlorophyll a content in plants exposed to *M. oryzae* CBMB20 were 10% higher than un-inoculated plants under non-stress conditions (Fig. 3A). However, there was no significant increase in chlorophyll b content under both conditions (Fig. 3B). Higher carotenoid contents in plants treated with *M. oryzae* CBMB20 under salt stress conditions showed 40% increase compared to control (Fig. 3C).

### Proline, Lipid Peroxidation, and Cellular Trehalose by *M. oryzae* CBMB20

**Fig. 4:**
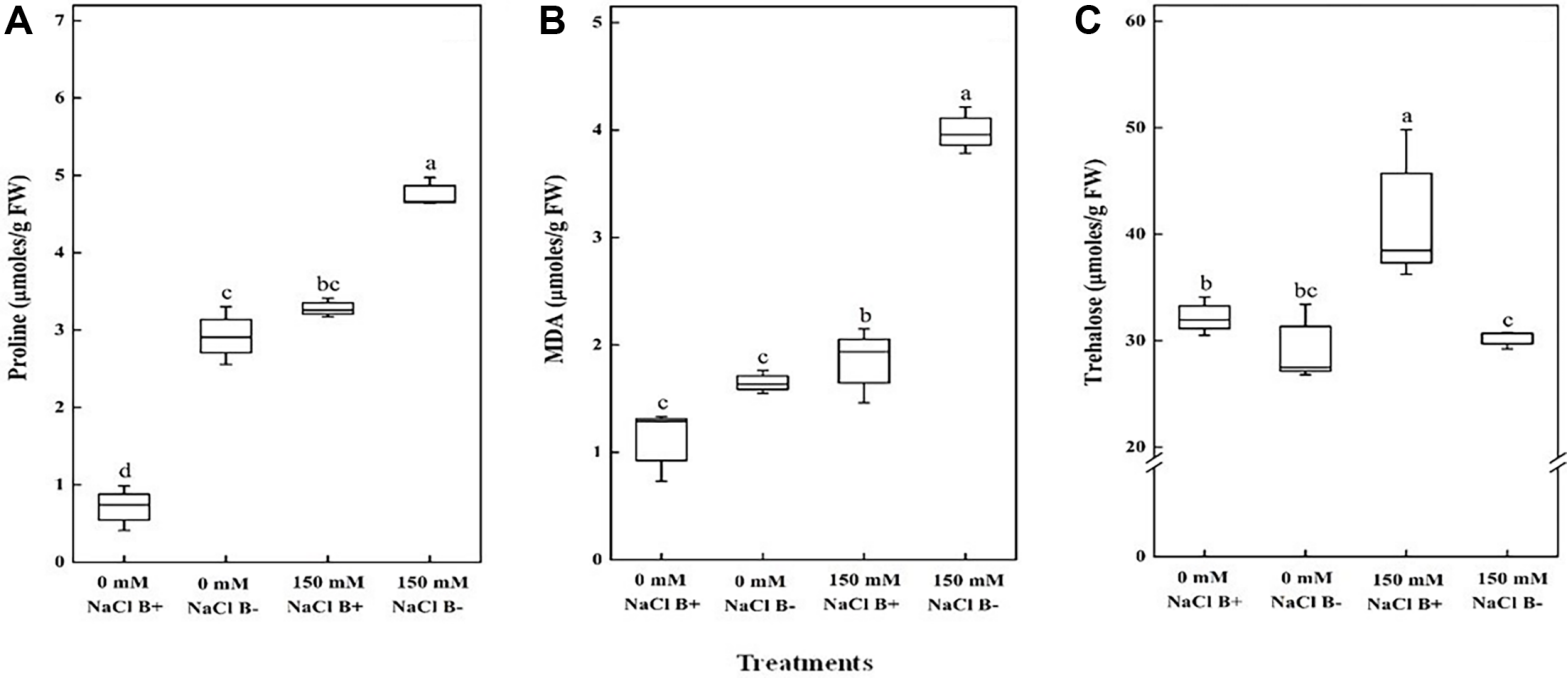
Effects of *M. oryzae* CBMB20 on (**A**) Proline content, (**B**) MDA content and (**C**) Cellular trehalose concentration of leaves in *Arabidopsis* plants grown under salt stress and non-stress conditions. The data represents the mean ± SD of three replicates and the different letters indicate the significant difference among treatments (*p* < 0.05); according to one way ANOVA test. Treatments details are the same as in Fig. 2.

The concentration of proline and MDA content were significantly increased in the plants when exposed to salt stress (Fig. 4). *Arabidopsis* plants under salt stress conditions showed higher proline content, the inoculation of plants had significantly (*p* < 0.001) lowered proline concentration compared to the non-inoculated plants under salt stress and non-stress conditions (Fig. 4A).

In this study, *M. oryzae* CBMB20 inoculation significantly lowered the malondialdehyde under salt stress conditions and MDA content increased significantly (*p* < 0.001) under high salinity in non-inoculated plants (Fig. 4B), indicating that *M. oryzae* CBMB20 plays a role in alleviating oxidative stress caused by high salinity. In both non-stress and salt stress conditions, plants exposed to *M. oryzae* CBMB20 accumulated higher trehalose (Fig. 4C). Accumulation of trehalose was significantly increased by *M. oryzae* CBMB20 application compared to the plants not treated with bacteria. However, there was no significant difference in trehalose production between inoculated and non-inoculated plants under non-stress conditions.

### Spatial Distribution of *M. oryzae* CBMB20 Strain in *Arabidopsis*

**Fig. 5:**
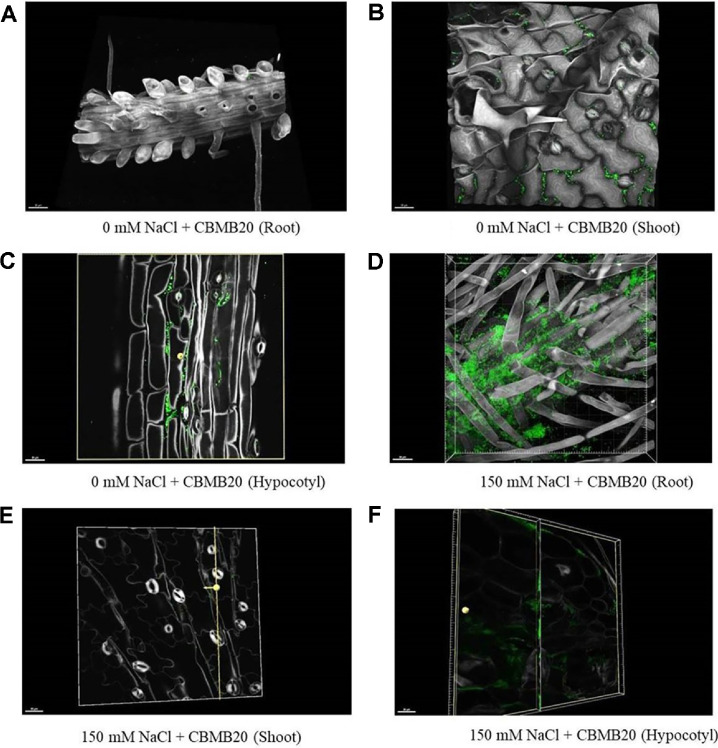
Confocal microscopic images of the persistence of GFP-tagged *M. oryzae* CBMB20 in roots, shoot and hypocotyl of *Arabidopsis thaliana*. (**A**) In root under non-stress condition, (**B**) in shoot under non-stressed condition, (**C**) in hypocotyl under non-stress condition, (**D**) in root under salt stress condition, (**E**) in shoot under salt stress condition, and (**F**) in hypocotyl under salt stress condition.

The extent of *M. oryzae* CBMB20 colonization in *A. thaliana* plantlets in roots and shoots was investigated using GFP-tagged *M. oryzae* CBMB20. Two weeks after seed treatment with bacterial suspension of GFP-expressing strain, seedlings exhibited GFP signals in various internal tissues of the plants such as roots, shoots and hypocotyl of *Arabidopsis* (Fig. 5).

### Exogenous Trehalose Application on Plant Growth

**Fig. 6:**
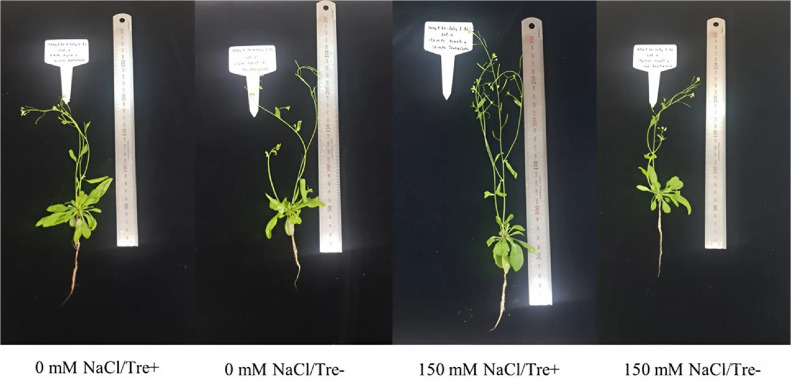
The growth of *Arabidopsis thaliana* plants after exogenous trehalose application under salt stress and non- stress conditions.

**Fig. 7:**
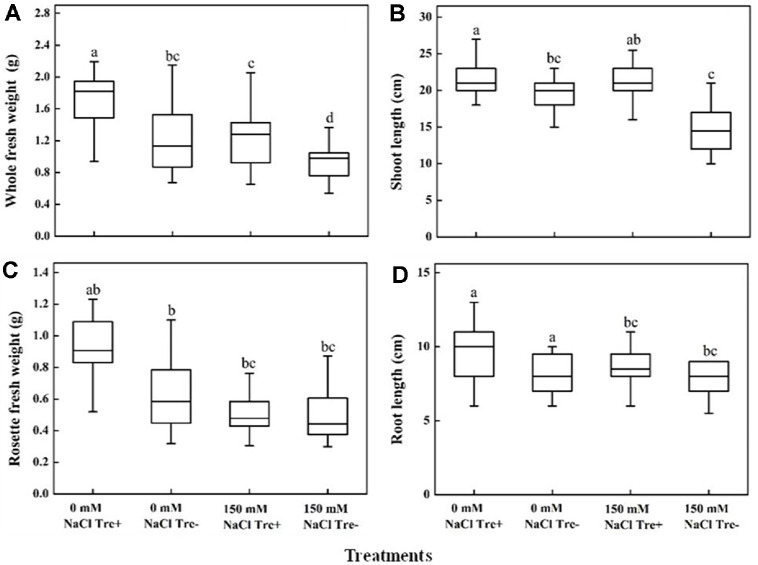
Effect of exogenous applied trehalose on plant growth under salt stress and non-stress conditions. *Arabidopsis* development phenotypes were analyzed in response to salt stress, application (Tre^+^) or without (Tre^-^) of 10 mM Trehalose under 0 mM NaCl and 150 mM NaCl concentration. (**A**) Whole Fresh Weight, (**B**) Shoot Length, (**C**) Rosette Fresh Weight, and (**D**) Root Length. Different letters indicate the significant difference among treatments (*p* < 0.05); according to one way ANOVA test; Data are the mean ± SD, *n* = 21.

Under both salt stress and non-stress conditions, exogenous application of trehalose positively impacted plant growth and development. In this study, exogenous application of trehalose significantly (*p* ≤ 0.05) alleviated the negative effects of salt stress by increasing shoot length in both stress and non-stress plants (Fig. 6). As shown in the figure, trehalose alleviated the symptoms of leaf chlorosis caused by salt stress. Application of trehalose increased fresh weight, shoot and root lengths in non-stress condition, but it’s not significant (Fig. 7). However, whole fresh weight and shoot length were significantly increased under stress conditions (Fig. 7A and 7B). The application of trehalose did not increase the rosette fresh weight and root length under salt stress condition (Fig. 7C and 7D).

### Exogenous Trehalose Application on Proline, Lipid Peroxidation, and Cellular Trehalose

**Fig. 8:**
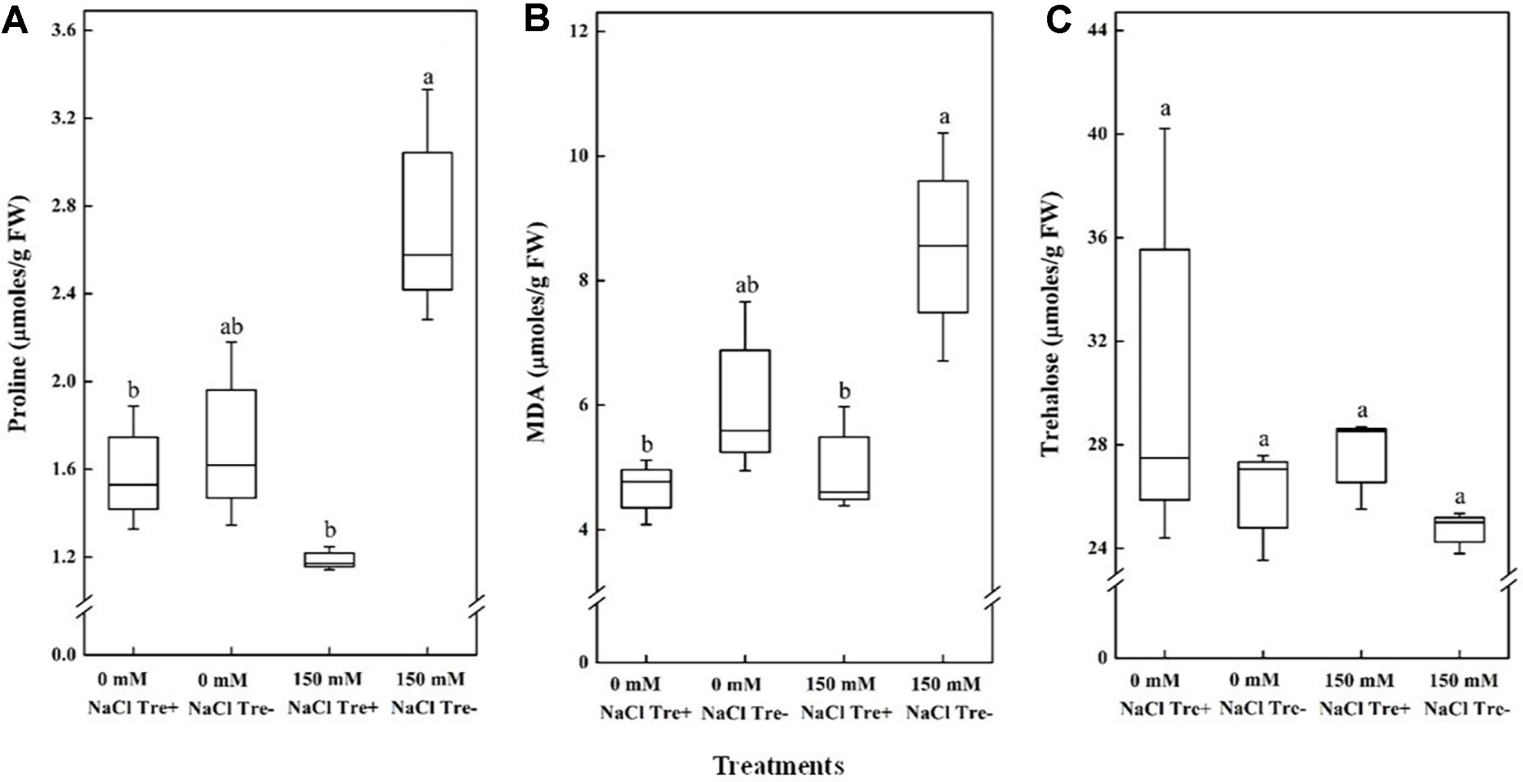
Effects of exogenous trehalose application on (**A**) Proline content, (**B**) MDA content, and (**C**) Cellular trehalose concentration of leaves in *Arabidopsis* plants grown under salt stress and non-stress conditions. The data represents the mean ± SD of three replicates and the different letters indicate the significant difference among treatments (*p* < 0.05); according to one way ANOVA test. Treatments details are the same as in Fig. 7.

Under non stress condition application of trehalose reduced proline and MDA content in *Arabidopsis* leaves (Fig. 8A and 8B). However, exogenous application of trehalose increased cellular trehalose content in plants (Fig. 8C), but the difference is not significant. Under salt stress condition plant proline and MDA was significantly (*p* < 0.05) reduced compared to untreated plants. Similar to non-stress condition exogenous application of trehalose increased cellular trehalose content in plants but the values are not significant.

## Discussion

Microbes associated to plants are generally prevalent and exhibit beneficial activity as PGPR by promoting plant growth, decreasing pathogensis, and mitigating stresses through a variety of underlying mechanisms. However, the exact process by which microorganisms mitigate stress differs depending on the kind and species of plant, and the degree of stress is still unknown. In this study, *A. thaliana* is used as a test plant in order to understand the mechanisms of salinity stress mitigation by *M. oryzae* CBMB20 and exogenous application of trehalose.

It was suggested that the PGP activity of certain bacteria depends on stress because some PGPR were shown to stimulate plant development under both normal and stressed environments, while others were only effective under stressful conditions and had no growth promotion effect under ideal conditions [40]. Results of this study showed the plant growth promoting ability of *M. oryzae* CBMB20 in non-stress and saline stress conditions. Plant growth promoting nature of the *M. oryzae* CBMB20 may rely on the production of phytohormones [41], ACC deaminase production [42] and enhanced photosynthetic traits [25]. Similar to this, *Methylobacterium* sp. 2A-inoculated plants outperformed non-inoculated plants in terms of biomass, root and shoot growth, and chlorophyll content in saline environments [43]. In line with this, bacterial inoculation dramatically decreased the negative impacts of stress, as seen by the significantly longer shoots of soybean plants [44]. Indeed, there have been many reports showing the stress mitigation by bacterial inoculation [45, 46].

As evidenced by the results, exogenous application of trehalose mitigated the negative effects of salt stress on growth parameters in *Arabidopsis*. These findings are consistent with previous studies, such as those conducted on rice [47], *Arabidopsis* [48], wheat [49] and maize [50]. Higher concentrations of trehalose were found to be supra-optimal and negatively impacted plant growth. Interestingly, the exogenous application of trehalose did not affect chlorophyll content in either non-stress or salt stress plants. In this study, the administration of trehalose enhanced plant growth, suggesting possible stress tolerance. Tomato growth was enhanced by exogenous trehalose, which also increased the plant's height, fresh mass, and relative water content of its leaves [28].

Leaf chlorophyll concentration serves as an indicator of salt tolerance. Chlorophyll degradation, caused by ions (such as NaCl or Cl) or ROS, leads to the deterioration of cell organelles, particularly in leaf tissue. It is clear that in saline environments, microbial inoculation can increase stomatal conductance and plants' availability of CO_2_. Specifically, one typical characteristic of microbe-primed plant salt tolerance is an increase in the amounts of chlorophyll and carotenoid in salt-stressed plants [51]. Inoculation with PGPR has been reported to enhance chlorophyll content in various plants compared to controls [52]. PGPR enhances photosynthesis through a number of ways, such as improved chlorophyll content accumulation [53] and increased plant water use efficiency (WUE) [54]. The pink-pigmented bacterium *M. oryzae* CBMB20 also produces carotenoids [55], which have strong redox characteristics and function as potent antioxidants [56].

It has been demonstrated that inoculation with ACC deaminase-containing bacteria significantly suppresses ethylene synthesis, thereby reducing chlorophyll degradation and preventing leaf senescence associated with elevated ethylene levels [57]. Inoculation of *M. oryzae* CBMB20 increased the chlorophyll content in *Arabidopsis* plants under salt stress condition than that of control plants. In line with this, use of *Methylobacterium* sp. 2A was found to improve the chlorophyll of potato plants grown under salt stress condition [43]. The quantity of chlorophyll in leaves dramatically dropped following treatment with salt stress. But after exogenous trehalose treatment, the content of chlorophyll improved.

Plant growth is inhibited by salinity stress, which also causes oxidative and osmotic stress, particularly ion (Na^+^ and Cl^−^ ion) toxicity. However, plants mitigate the consequences of salt stress in a number of ways, including the production of suitable solutes (osmolytes), the activation of an antioxidant defense system, and the adaptive regulation of hormones connected with stress. Osmotolerance is achieved through the accumulation of compatible solutes, which protect cells from salt-induced damage [58].

Proline is well known as a compatible solute and is a key compound in assessing salt tolerance [59]. Proline builds up as a solute in plants and is essential for a number of physiological functions, such as protein solvation and structural stability, membrane integrity preservation, lipid membrane reduction and oxidation, reactive oxygen species scavenging, and buffering of cellular redox potential under stress. In the present study *M. oryzae* CBMB20 inoculation reduced the proline content. Proline accumulation in plants is the first response to stress in order to protect cells from injury. It has been reported that proline enhances plant tolerance to salt stress by not only regulating osmotic pressure but also stabilizing various proteins, enzymes, and cell membranes [60]. Osmolytes help prevent cell membrane disruption and enhance membrane stability under salt stress conditions.

In contrast, proline levels were significantly lower in plants exposed to bacteria compared to those that were not. Other studies have also reported that PGPR reduce proline content. The physiological importance of proline accumulation in salt-stressed plants is controversial and the effects of microbial inoculations on plant proline accumulation during saline stress also differ [51]. For example, the proline levels in *Arachis hypogaea* shoots inoculated with six PGPR strains were markedly reduced under salt stress conditions [61]. As salt concentration increased, the proline content in rice also increased. However, rice inoculated with PGPR exhibited lower proline levels compared to non-inoculated plants [62]. Additionally, *Bacillus amyloliquefaciens* SQR9 inoculation decreased proline levels in maize under salt stress conditions [63]. It is possible that plants treated with PGPR experienced less severe salt stress, resulting in lower proline accumulation compared to untreated plants [61].

Exogenous application of trehalose reduced the salinity-induced damage in the plants by regulating the levels of osmotic substances. Proline has been reported to assist with osmotic adjustment under stress conditions [64]. Since both proline and cellular trehalose act as osmoprotectants, the decrease in proline accumulation observed with exogenous trehalose treatment under salt stress suggests either a compensatory role for trehalose or a reduced need for proline. Consistent with previous reports, exogenous trehalose was found to lower proline levels in plants under salt stress [65]. Another recent study found that, exogenous trehalose application decreased proline content in wheat plants under salt stress condition [66]. Plants use defensive adaptation mechanisms, such as osmotic regulation or osmoprotection, to survive in challenging environments.

MDA concentration is used as a gauge to ascertain the plant's redox state and directly correlated with oxidative damage to the cell membrane in plants [67]. In the present study, *Arabidopsis* plants subjected to salt stress exhibited reduced MDA levels when inoculated with *M. oryzae* CBMB20. In accord with, both liquid and chitosan-immobilized inoculation *M. oryzae* CBMB20 cells showed promise in reducing tomato plant salt stress by registering lower MDA level [19]. Lower MDA concentrations are indicative of reduced membrane damage or enhanced salt tolerance in plants [7, 68]. When compared to other treatments, *Pseudomonas* spp. inoculation dramatically reduced the MDA concentration in red pepper under salt stress conditions [69].

MDA levels indicate the extent of lipid peroxidation in cell membranes, which is closely associated to the plant's effective enzymatic clearance systems [70]. Previous studies have demonstrated that exogenous trehalose positively reduced MDA levels under stress conditions [71-73]. These studies showed that high MDA accumulation indicated membrane lipid peroxidation, which is typically lower in stress-resistant plants. As observed in this study, MDA concentration dramatically increased following salt treatment, suggesting that salt stress severely damaged plant cell membranes and caused a high production of reactive oxygen species and free radicals that damaged the membrane. Following exogenous trehalose treatment, the MDA content decreased. A recent report showed that treatment of wheat seeds with trehalose significantly reduced the MDA content under salt stress [66]. In the present study, trehalose reduced MDA levels in plants, highlighting its role as a protective agent for membranes under abiotic stresses [74, 75].

Trehalose is a disaccharide that works in concert with other antioxidant apparatuses in plant cells to shield cells from ROS during times of stress [76]. Salinity stress resulted in a significant increase in cellular trehalose content in *Arabidopsis* plants compared to controls with bacterial inoculation. Cellular trehalose production can serve as a stress-reducing strategy, as it supports plant growth under both drought and high salt conditions [4]. Higher concentrations of cellular trehalose have been found in the root nodules of *Phaseolus vulgaris* and *Medicago truncatula* in response to drought and salt stress. This indicates the crucial role of cellular trehalose in signaling during plant-bacteria interactions, enhancing plant yield, growth, and adaptation to harsh conditions [77]. Furthermore, the enhancement of tomato root development and growth under saline conditions was totally hindered by the mutation of cellular trehalose synthesis in *Pseudomonas putida* UW4, indicating a crucial function for osmolytes produced by microbes in plant salt tolerance [78].

Exogenous application of trehalose did not have any impact on the endogenous concentration of trehalose in the present study. This is in concordance with previous results, which also reported no significant change in cell trehalose levels following exogenous application [79]. According to Redillas [80], exogenously administered trehalose boosts the activity of defense-response proteins in crops and enhances rice plants' resilience to salty environments [65].

Root colonization by inoculated bacteria is essential for establishing beneficial interactions between bacteria and their host plants [81, 82]. Linking reporter genes, such as GFP, to plasmids used for transforming specific bacteria has greatly improved our understanding of rhizobacteria localization within plants. It is important to note that GFP-labeled cells were observed inside the roots of *A. thaliana* treated with the GFP-labeled strain, indicating that the strain colonizes the roots effectively. An endophyte successfully colonized the roots of various plant species, such as *Arabidopsis* [6], corn [83] and switch grass [84]. Overall, *M. oryzae* CBMB20 not only promoted plant growth and effectively colonized root tissues but also mitigated the adverse effects of salt stress. This strain enhances salt stress tolerance in *A. thaliana*, enabling the plants to thrive under stressful conditions. The study demonstrated that the GFP technique effectively assesses the colonization ability of various rhizobacterial species, though it may not apply to all species.

## Conclusion

This study indicates that trehalose may function as an elicitor of biological processes, and that plant growth-promoting bacteria could be a valuable tool for agriculture in saline regions, potentially contributing to global food security and agricultural sustainability. These results demonstrated the potential applications of trehalose and *M. oryzae* CBMB20 in plant stress management; however, more investigation is required to assess CBMB20's suitability for agronomic management in open-field settings.
